# Enhanced Microwave Absorption Properties of α-Fe_2_O_3_-Filled Ordered Mesoporous Carbon Nanorods

**DOI:** 10.3390/ma6041520

**Published:** 2013-04-15

**Authors:** Yiming Wang, Liuding Wang, Hongjing Wu

**Affiliations:** Department of Applied Physics, School of Science, Northwestern Polytechnical University, Xi’an 710072, China; E-Mails: wym2010@mail.nwpu.edu.cn (Y.W.); wangld@nwpu.edu.cn (L.W.)

**Keywords:** ordered mesoporous carbon, α-Fe_2_O_3_, dielectric loss, microwave absorption

## Abstract

A novel kind of α-Fe_2_O_3_-filled ordered mesoporous carbon nanorods has been synthesized by a facial hydrothermal method. Compared with dendritic α-Fe_2_O_3_ micropines, both a broader effective absorption range—from 10.5 GHz to 16.5 GHz with reflection loss (RL) less than −10 dB—and a thinner matching thickness of 2.0 mm have been achieved in the frequency range 2–18 GHz. The enhanced microwave absorption properties evaluated by the RL are attributed to the enhanced dielectric loss resulting from the intrinsic physical properties and special structures.

## 1. Introduction

In recent years, the study of microwave absorbing materials has attracted much attention for electromagnetic interference (EMI) shielding and electromagnetic (EM) wave absorbing applications, for both commercial and defense purposes [1,2,3]. Traditional spinel-type ferrites have been extensively studied, however they have some disadvantages such as low permeability, caused by Snoek’s limit, and large density hinder their further development [4]. On the other hand, dielectric absorbers have potential as microwave absorbents due to advantages such as low density, controllable dielectric loss ability, and so on [5]. However, compared with ferrite materials, dielectric fillers such as carbon fiber and multi-walled carbon nanotubes are used to control complex permittivity only [6]. The complex permeability and permittivity of dielectric dissipation materials are out of balance, and their impedance matching characteristic is poor [7]. Recently, many studies have been carried out to investigate the complementary behavior of magnetic or dielectric microwave absorber coatings by mixing multi-walled carbon nanotubes or metallic magnetic materials [8,9]. Several groups have reported excellent microwave absorption properties of heterogeneously structured nanocomposites, such as Fe_3_O_4_-poly(3,4-ethylenedoxythiophene) (PEDOT) microspheres [10], hierarchical magnetic yolk/shell microspheres [11], nanocomposites, *etc.* These heterogeneous structures exhibit interesting chemistry as well as effective interfaces and material-dependent properties, endowing such structures with enhanced properties, or satisfactory synergistic effects for microwave absorption enhancement. However, up until now very little attention has been directed toward the magnetic particle-filled ordered mesoporous carbon materials for microwave absorption applications.

The purpose of this study was to investigate the electromagnetic and microwave absorption properties of hybrid nanorods consisting of ordered, mesoporous carbon (OMC) and α-Fe_2_O_3_, which may adjust the dielectric properties of α-Fe_2_O_3_and OMC and contribute to the microwave absorption properties. Most interestingly, it exhibited a broader absorption bandwidth and lighter weight in the studied range of 2–18 GHz than the as-synthesized dendritic α-Fe_2_O_3_ micro-pines. It was found that a multi-loss combination in the unique α-Fe_2_O_3_/OMC hybrid structures contributed to enhanced microwave absorption.

## 2. Results and Discussion

**Figure 1:**
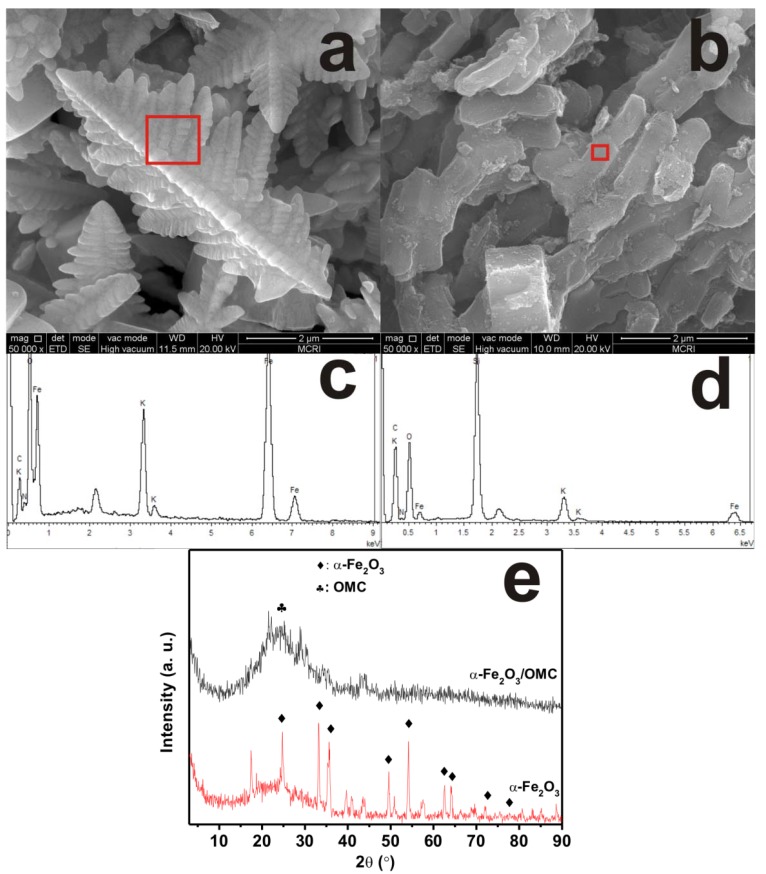
Structural characterizations of dendritic α-Fe_2_O_3_ micropines and α-Fe_2_O_3_/OMC nanorods: (**a**,**b**) scanning electron microscope (SEM) images; (**c**,**d**) energy dispersive spectroscopy (EDS) spectrum; and (**e**) X-ray diffraction on a diffractometer (XRD) patterns.

Representative SEM images of the as-synthesized products are shown in Figure 1a,b respectively. It can be clearly seen that dendritic Fe_2_O_3_ micropines can be formed by using a facial hydrothermal reaction at low temperatures [12]. As shown in Figure 1a, the high magnification image of a single dendrite shows a hierarchical structure with tertiary branches. The lengths of the dendrite trunks are 3–5 μm, and those of the branch trunk range from 50 nm to 1 μm, which is very consistent with the report of Sun *et al.* [12]. When OMC is added to the solution of 0.1 mol L^−1^ K_3_[Fe(CN)_6_] in a Teflon-sealed autoclave, Fe_2_O_3_-filled OMC structures are obtained, as shown in Figure 1b. Compared with the electron microscopy of undoped OMC [13], it is found that the Fe_2_O_3_-filled OMC structures are relatively thick, suggesting that Fe_2_O_3_ nanoparticles are mostly embedded in the carbon walls of OMC. Figure 1c,d show the main peaks of O, Fe, and C elements in the energy dispersive spectroscopy (EDS) spectrum, indicating that the nanorods contain a Fe_2_O_3_ component. Figure 1e shows the XRD pattern of the dendritic Fe_2_O_3_ micropines, in which all diffraction peaks indicated by Miller indices can be indexed to α-Fe_2_O_3_. There are also peaks which are not well defined. These peaks may be assigned to the intermediate products when preparing dendritic Fe_2_O_3_ micropines, which would not contribute to the absorption properties of the composites. It suggests that α-Fe_2_O_3_ dendrites are highly crystalline. However, the α-Fe_2_O_3_-filled OMC nanocomposites are relatively amorphous, exhibiting weak diffraction peaks for α-Fe_2_O_3_ in the XRD pattern, expect for a diffraction hump of carbon around 15°–35°.

**Figure 2:**
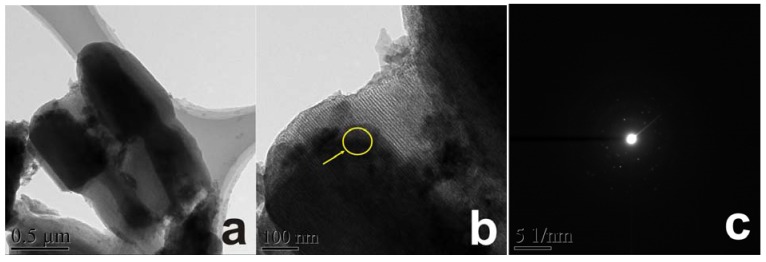
(**a**,**b**) transmission electron microscope (TEM) images and (**c**) SAED of α-Fe_2_O_3_/OMC nanorods.

The morphology of as-prepared α-Fe_2_O_3_/OMC hybrid structure was investigated in detail by TEM, and shown in Figure 2. Figure 2a shows a low magnification TEM micrograph of α-Fe_2_O_3_/OMC hybrid nanorods, in which all the nanorods seem to have a core-shell structure. However, the α-Fe_2_O_3_ core regime is formed by filling the mesopores of OMC with α-Fe_2_O_3_ nanoparticles. The formation of the OMC shell may have been caused by not completely filling the α-Fe_2_O_3_ nanoparticles. Strictly, it is not really a core-shell structure. As shown in Figure 2b, the α-Fe_2_O_3_ appears black and OMC is light colored in the image, because hematite has a higher mass–thickness contrast. The bright dot patterns from SAED of the sample given in Figure 2c can be well-indexed to the crystalline phase of hematite.

**Figure 3:**
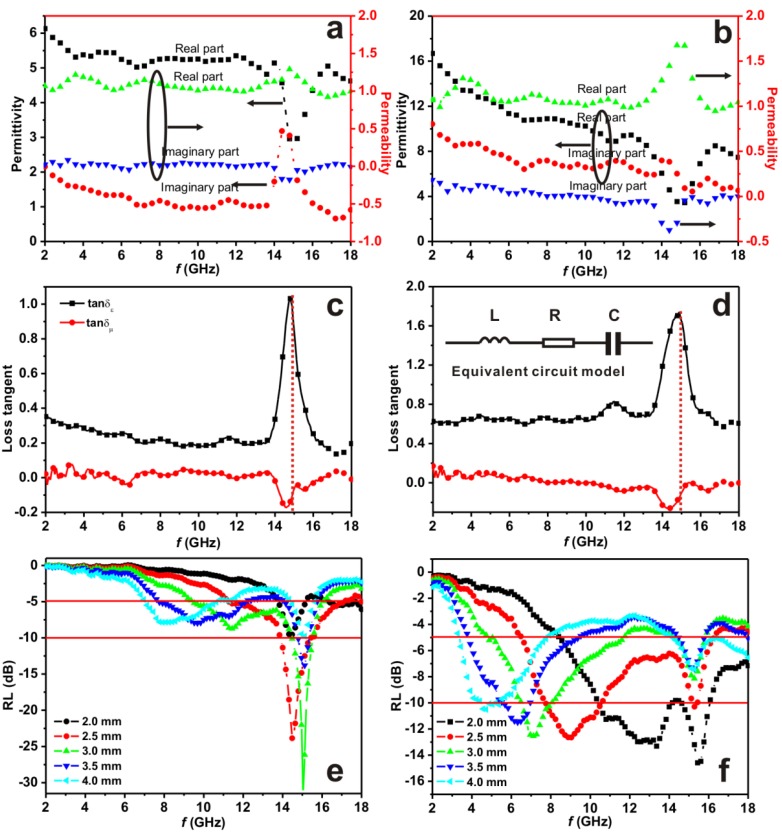
(**a**,**b**) relative complex permittivity and permeability; (**c**,**d**) dielectric and magnetic tangent loss; (**e**,**f**) reflection loss of α-Fe_2_O_3_ microdendrites and α-Fe_2_O_3_/OMC nanorods respectively. The inset in Figure 3d is a *LRC* equivalent circuit model.

The electromagnetic (EM) parameters (relative complex permittivity, *ε* = *ε*′ − *jε*′′ and relative complex permeability, *μ*_r_ = *μ*′ − *jμ*′′) of the wax composites containing 50 wt % of the as-synthesized samples were measured at room temperature. Figure 3a,b show the measured *ε*_r_ and *μ*_r_ in the range of 2–18 GHz for the two samples. The *ε*′ and *ε*′′ values of α-Fe_2_O_3_ microdendrites are in the range 3–6 and 0.5–3 over a frequency region of 2–18 GHz. However, those values are much lower than those of α-Fe_2_O_3_/OMC hybrid nanorods (4–17 for *ε*′ and 4.5–11.5 for *ε*′′). It suggests that α-Fe_2_O_3_/OMC hybrid nanorods have a strong dielectric loss when exposed to EM waves. The dielectric loss tangent ($\text{tan}\;\delta_{e}=\varepsilon \prime \prime /\varepsilon \prime$) of the two samples is shown in Figure 3c,d. The $\text{tan}\;\delta_{e}$ values of α-Fe_2_O_3_/OMC hybrid nanorods larger than 0.6 are distributed between 2 and 18 GHz and exhibit a peak value of 1.7 at 15 GHz, whereas the values for α-Fe_2_O_3_ microdendrites are in the range 0.2–0.4 over the frequency range 2–14 GHz, indicating a strong dielectric resonance at the high frequency range in the case of α-Fe_2_O_3_/OMC hybrid nanorods. In general, high dielectric loss results from the inherent physical properties of OMC and their special heterogeneous structures. Ordered mesoporous carbon (OMC) materials have large surface areas, so the number of surface atoms with unsaturated bonds is greatly increased, resulting in an increase of dipoles. Consequently, the dipole polarizations could contribute to dielectric loss. Furthermore the microstructure of α-Fe_2_O_3_ nanoparticles filling a carbon matrix would provide additional interfaces around the nanoparticles, and the interfacial polarization associated with relaxation could give rise to dielectric loss as well. The above two dielectric loss aspects can be confirmed by the *Cole-Cole* semicircle, as shown in Figure 2S(a) and Figure 2S(b) [13,14,15], because the *Cole-Cole* semicircle is a typical feature of dielectric relaxation. In addition, because of the addition of the conductive material OMC, the increase in electric conductivity of the α-Fe_2_O_3_/OMC hybrid nanorods results in an enhanced conductance loss, as shown in Figure 2S(a) and Figure 2S(b) (red dashed lines) [16,17].

The *μ*′ and *μ*′′ values of the two samples (Figure 3a,b) indicate that *μ*′ is close to one, while the *μ*′′ is almost equal to zero in the frequency range 2–18 GHz, expect for a resonant frequency at around 15 GHz. Moreover, the *μ*′′ value is negative at this resonant frequency. Considerable attempts have been made to obtain an exact solution of the phenomenon that causes the values of permeability *μ*′′ to be negative at some frequencies. Chiu [18] has pointed out that it is meaningless and may be due to noise, although we have previously explained the phenomenon as the outward radiation of magnetic energy, resulting in a conversion of the incident wave to other forms of energy [13,14,15]. So, in the present work we consider that this phenomenon may be related to the eddy currents on the surface of α-Fe_2_O_3_/OMC structures and α-Fe_2_O_3_ dendrites which can produce an induced magnetic field, and in turn radiate EM waves, leading to an increase in the total magnetic energy and hence a negative *μ*′′ in the measured frequency range [15]. According to the references [13,14,15], if the values of $\mu \prime \prime {\left(\mu \prime \right)}^{-2}f^{-1}$ is kept constant while the frequency is varied, the magnetic loss only results from eddy current loss. From Figure 2S(c) and Figure 2S(d) it can be found that an eddy current may occur on the surface of α-Fe_2_O_3_/OMC composites. The magnetic loss tangent ($\text{tan}\;\delta_{m}=\mu \prime \prime /\mu \prime$) of the two samples is simply the inverse to that of $\text{tan}\;\delta_{e}$, as shown in Figure 3c,d, which can be explained by the *LRC* equivalent circuit model (see the inset in Figure 3d), where *L*, *R*, and *C* are the inductance, resistance and capacitance respectively. In particular, the eddy current effect regarded as inductance, the dielectric relaxation polarization playing a role as capacitance, and the conductance loss acted as resistance all contribute to the *LRC* equivalent circuit model. The inverse change trend is attributed to the capacitance leading or lagging behind the inductance by an angle of 90° [15].

**Figure 4:**
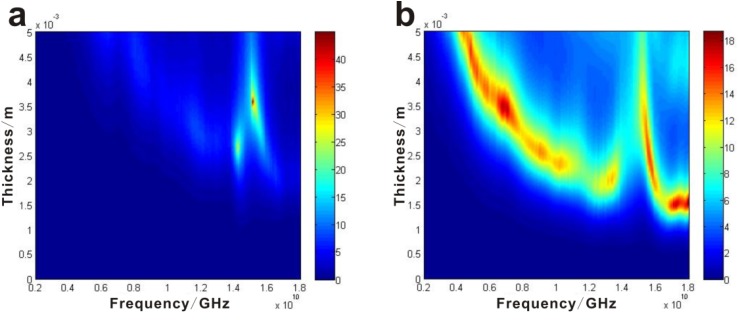
Reflection loss map of (**a**) α-Fe_2_O_3_ microdendrites and (**b**) α-Fe_2_O_3_/OMC nanorods.

On the basis of transmission line theory [19] the reflection loss (RL) can be calculated; the results are shown in Figure 3e,f. It can be seen clearly that the α-Fe_2_O_3_/OMC hybrid nanorods with a coating layer thickness of 2.0 mm exhibit a broader absorption bandwidth than the α-Fe_2_O_3_ microdendrites, corresponding to an RL below −10 dB with the same thickness, which can reach up to 6.0 GHz (from 10.5 GHz to 16.5 GHz). It is also noted that the absorption peak value of α-Fe_2_O_3_ microdendrites is much higher than that of the α-Fe_2_O_3_/OMC hybrid nanorods, but its absorption bandwidth is too narrow to be widely applied. Figure 4a,b show the color map of the reflection loss values calculated from the measured EM parameters of nanocomposites. It is obvious that for α-Fe_2_O_3_/OMC hybrid nanorods, RL exceeds −10 dB in the range of 4–18 GHz for absorber thicknesses of 1.5–5.0 mm. Compared with the recent representative nanocomposites, the as-synthesized α-Fe_2_O_3_/OMC hybrid nanorods present a similar microwave absorption performance with thin thickness and broad absorption range, as shown in Table 1. However, we report a novel synthetic approach that can be used to prepare magnetic particle-filled ordered mesoporous carbon materials for microwave absorption application. The enhanced microwave properties of the α-Fe_2_O_3_/OMC hybrid nanorods are attributed to a major dielectric loss and a minor eddy current loss, leading to the microwave absorption enhancement.

**Table 1:** Microwave absorption performance of some representative nanocomposites.

Samples	Minimum RL value (dB)	Frequency range (GHz) (RL < −10 dB)	*d*_m_ (mm) (RL < −10 dB)	Percentage (wt % or vol %)	References
Fe_3_O_4_/PEDOT	−30	~8–13	4 mm	20 (vol %)	[10]
Porous Fe_3_O_4_/carbon	−27.9	~13–18	2 mm	55 (wt %)	[16]
α-Fe_2_O_3_	−23.9	13.8–15.5	2.5 mm	50 (wt %)	this work
α-Fe_2_O_3_/OMC	−15.0	10.5–16.5	2 mm	50 (wt %)	this work

**Figure 5:**
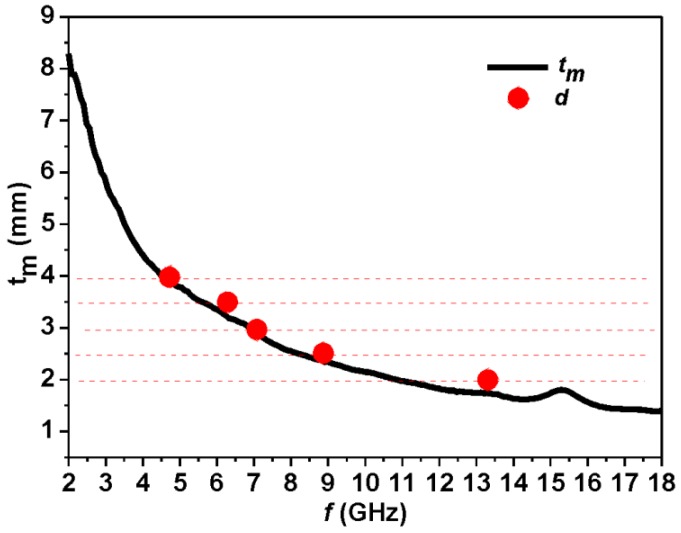
The frequency dependence of matching thickness *d* and calculated thickness *t_m_* for α-Fe_2_O_3_/OMC hybrid nanorods.

Besides the major dielectric loss, electromagnetic waves could be absorbed via a so-called “geometrical effect” [17]. The “geometrical effect” means that when the thickness of absorber (*t_m_*) satisfies the following Equation (1), the reflected waves are totally canceled in the air-absorber interface:

(1) $$t_{m}=\frac{n\lambda_{m}}{4},\left(n=1,3,5...;\lambda_{m}=\frac{\lambda_{0}}{\sqrt{\left|\varepsilon_{r}\mu_{r}\right|}};\lambda_{0}=\frac{c}{f_{m}}\right)$$

where $\lambda_{m}$ is the wavelength in the absorber at some specific frequency, *f*_m_; $\left|\varepsilon_{r}\right|$ and $\left|\mu_{r}\right|$ are the moduli of $\varepsilon_{r}$ and $\mu_{r}$ respectively; and $\lambda_{0}$ is the wavelength in free space. The calculated frequency dependence of *t_m_* is shown in Figure 5 (black line) for the α-Fe_2_O_3_/OMC hybrid nanorods sample. The thickness (denoted as *d*)—corresponding to the minimum RL in Figure 4f—is also plotted in Figure 5 with red circles. The calculated thickness *t*_m_ is consistent with *d*, which implies that the corresponding *f*_m_ and the matching thickness *d* is coincident with the quarter wavelength matching condition. Therefore, the above calculations justify that the selective absorption properties of the α-Fe_2_O_3_/OMC hybrid nanorods at certain frequencies is due to the geometric effect.

## 3. Experimental Section

### 3.1. Materials

The synthesis of mesoporous silica (*i.e.*, SBA-15) was prepared using the triblock copolymer, EO_20_PO_70_EO_20_ (Pluronic P123, BASF) as the surfactant and tetraethylorthosilicate (TEOS, 98%, Acros, Japan) as the silica source. Raw commercial HCl, H_2_SO_4_, NaOH, K_3_[Fe(CN)_6_], and sucrose as the carbon source were purchased from Sinopharm Chemical Reagent Co. Ltd., China.

### 3.2. Sample Preparation

In this work, OMC was synthesized by using mesoporous silica SBA-15 as a hard template, as reported in our previous works [13,14,15]. One-dimensional (1-D) α-Fe_2_O_3_/OMC hybrid nanorods were synthesized by a facile hydrothermal method in a 100 mL autoclave at 140 °C for 48 h and masses of 3.29 g for K_3_[Fe (CN)_6_] and 0.50 g for OMC were chosen. For comparison, dendritic α-Fe_2_O_3_ micropines were fabricated by the same method, except for the addition of OMC. Then the resulting compounds were filtrated, washed and dried in air at 80 °C overnight. The paraffin wax composites were prepared by ultrasonic agitation to mix a certain weight fraction (50 wt %) of absorbent with paraffin wax matrix.

### 3.3. Measurements

The as-prepared samples were characterized by X-ray diffraction on a diffractometer (XRD; D/MAX, Rigaku, Japan), scanning electron microscope (SEM; JSM-5610LV, JEOL, Japan), energy-dispersive X-ray spectroscopy (EDS; OXFORD INCA, UK), and transmission electron microscope (TEM; JEM-3010, JEOL, Japan). The as-prepared paraffin wax composites were pressed into a toroid with an outer diameter of 7.0 mm, inner diameter of 3.04 mm, and thickness of 3.0 mm. The scattering parameters of the toroidal samples (S_11_, S_21_) were measured by bidirectional transmission/reflection method in a coaxial measurement fixture (as shown in Figure 1S) with the HP 8720B network analyzer over 2–18 GHz. The electromagnetic parameters—relative complex permittivity, *ε_r_* = *ε*′ − *jε*′′ and relative complex permeability, *μ*_r_ = *μ*′ − *jμ*′′—were determined by the complex scattering parameters using the Nicolson–Ross model [20,21]. The reflection loss (RL) was calculated using the following equations [19]:

(2) $$Z_{in}=Z_{0}{\left(\mu_{r}/\varepsilon_{r}\right)}^{1/2}\text{tanh}[j(2\pi fd/c){\left(\mu_{r}\varepsilon_{r}\right)}^{1/2}],\;RL=20\log|(Z_{in}-Z_{0})/(Z_{in}+Z_{0})|$$

where $\mu_{r}$ and $\varepsilon_{r}$ are the relative permeability and permittivity, *f* is the frequency of the electromagnetic wave, *d* is the thickness of the absorber, *c* is the velocity of light, $Z_{0}$ is the impedance of free space, and $Z_{in}$ is the input impedance of the absorber.

## 4. Conclusions

In summary, one dimensional (1D) α-Fe_2_O_3_/OMC hybrid nanorods have been fabricated by the facial hydrothermal method. The unique heterogeneous structure of α-Fe_2_O_3_/OMC hybrid nanorods greatly enhance the dielectric loss of the materials, and therefore not only a broader effective absorption range from 10.5 GHz to 16.5 GHz with reflection loss less than −10 dB, but also a lighter weight and thinner matching thickness (2.0 mm) have been achieved. Combining the complementarities with intrinsic physical properties and special structures, the hybrid nanorods can be used as a promising EM wave absorber.

## Supplementary Materials

Supplementary File 1
