# Supplementary material for: Enhanced Microwave Absorption Properties of α-Fe2O3-Filled Ordered Mesoporous Carbon Nanorods
Source: Materials (Basel). 2013 Apr 15;6(4):1520–9. doi: 10.3390/ma6041520 (PMC5452305; doi:10.3390/ma6041520)
Supplement: Supplementary File 1 [file materials-06-01520-s001.pdf]

Supplementary Information

Figure S1. Two-port coaxial fixture used in measuring the complex scattering parameters (S_{11}, S_{21}).

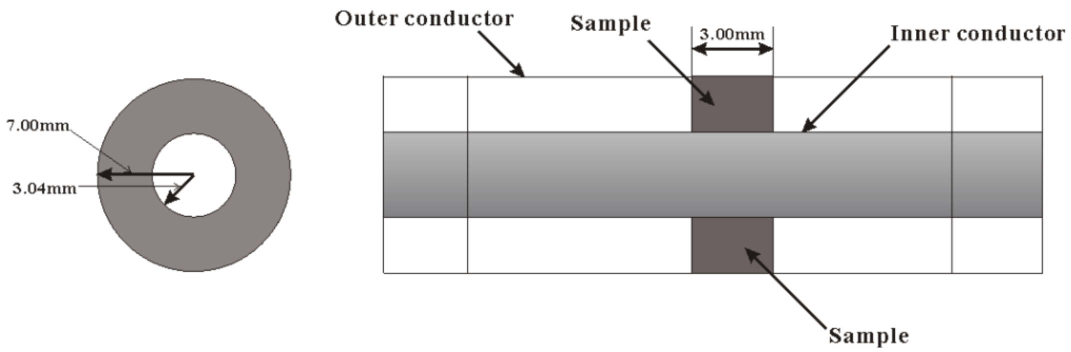

Figure S2. (**a**,**b**) *Cole-Cole* semicircles and (**c**,**d**) values of $\mu''(\mu')^{-2} f^{-1}$ as a function of frequency for α-Fe_2O_3 microdendrites and OMC@α-Fe_2O_3 core-shell nanorods, respectively.

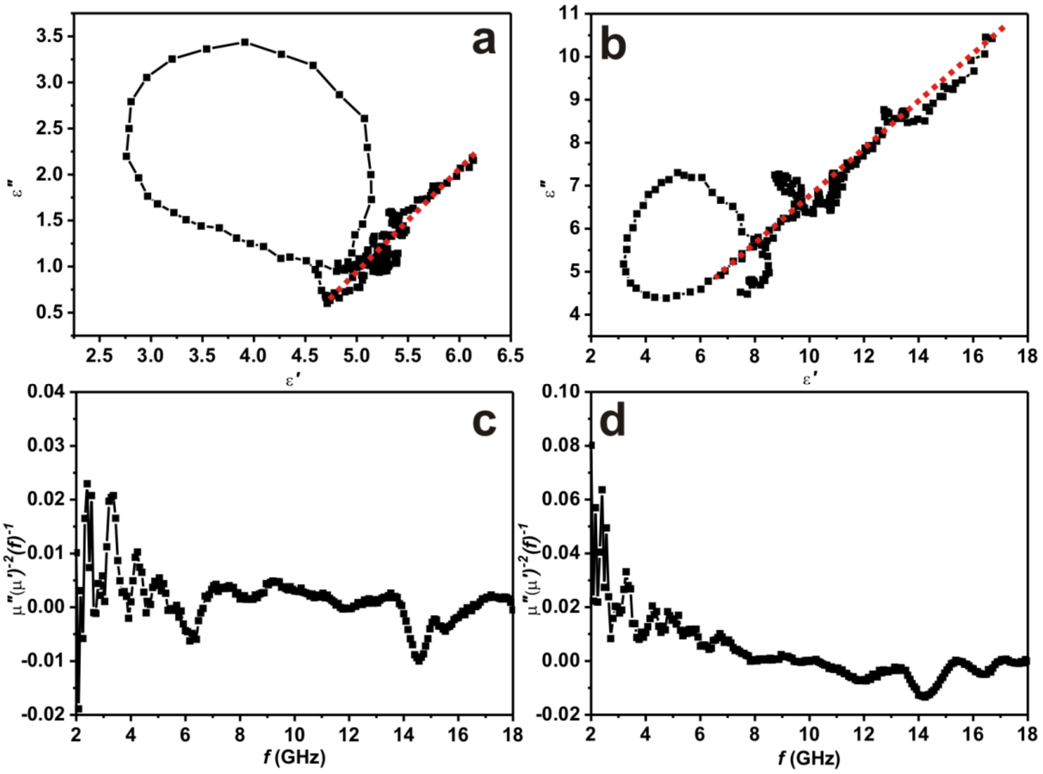

© 2013 by the authors; licensee MDPI, Basel, Switzerland. This article is an open access article distributed under the terms and conditions of the Creative Commons Attribution license (http://creativecommons.org/licenses/by/3.0/).
